# Comparative Assessment of Cortisol, Leptin, Haptoglobin and Inflammatory Interleukins in Modicana and Holstein Cows During the Peripartum Period

**DOI:** 10.3390/ani16172711

**Published:** 2026-09-01

**Authors:** Federica Arrigo, Maria Rizzo, Giulia Sisia, Claudia Giannetto, Elisabetta Giudice, Giuseppe Piccione, Francesca Arfuso

**Affiliations:** Department of Veterinary Sciences, University of Messina, 98121 Messina, Italy; federica.arrigo@studenti.unime.it (F.A.); giulia.sisia@studenti.unime.it (G.S.); claudia.giannetto1@unime.it (C.G.); elisabetta.giudice@unime.it (E.G.); giuseppe.piccione@unime.it (G.P.); francesca.arfuso@unime.it (F.A.)

**Keywords:** peripartum period, dairy cows, stress, native breed, Modicana, Holstein

## Abstract

Understanding breed-related physiological responses during the peripartum period may contribute to the development of more resilient and sustainable dairy systems. This study compared endocrine and inflammatory responses in a native Sicilian breed, Modicana, and a high-producing cosmopolitan breed, Holstein, during the 15 days before and 15 days after calving. Twenty cows (10 per breed), managed under the same conditions, were monitored for serum cortisol, haptoglobin, leptin, IL-1β, IL-6 and TNF-α. Both breeds showed peripartum changes in cortisol and haptoglobin concentrations (*p* < 0.0001). Holstein cows showed higher cortisol concentrations before calving and at calving, as well as higher haptoglobin concentrations at calving. Leptin concentrations were consistently higher in Holsteins (*p* < 0.001), whereas Modicana cows showed a transient prepartum decline. The inflammatory biomarkers also showed breed- and time-dependent patterns, with differences observed in IL-1β, IL-6, and TNF-α. Overall, the two breeds showed different endocrine and inflammatory responses during the peripartum period. These findings suggest possible breed-related differences in the physiological response to the transition period, although physiological resilience was not directly assessed.

## Simple Summary

This study compared the endocrine and inflammatory responses of Modicana, a native Sicilian dairy breed, and Holstein cows, a high-producing cosmopolitan breed, during the peripartum period. Twenty cows were enrolled, including 10 cows of each breed, and serum concentrations of cortisol, haptoglobin, leptin, IL-1β, IL-6, and TNF-α were evaluated at different time points before and after calving. Holstein and Modicana cows showed different patterns of endocrine and inflammatory biomarkers during the peripartum period. Overall, the observed breed-specific differences suggest that Modicana and Holstein cows may differ in their physiological responses to the transition period.

## 1. Introduction

The peripartum period represents one of the most physiologically complex and delicate stages in a cow’s life. During this time, the animal must undergo profound metabolic, endocrine, and immune adjustments to cope with calving and the onset of lactation [1]. The transition from pregnancy to lactation is accompanied by a marked increase in energy requirements associated with colostrum and milk synthesis [2]. This increased demand can promote the mobilization of body reserves and activate metabolic and immune regulatory mechanisms which, when insufficiently compensated, may increase the risk of metabolic, inflammatory, and reproductive disorders [3,4]. Among the endocrine factors involved in these adaptations, leptin plays a key role in the regulation of energy balance. In addition to its metabolic functions, leptin also exerts immunomodulatory effects, influencing lymphocyte activity and the production of inflammatory mediators [5,6,7]. At the same time, the physiological stress response during the peripartum period is orchestrated by the activation of the hypothalamic−pituitary−adrenal (HPA) axis. Following stressful circumstances, including the peripartum period, the HPA axis is activated to restore homeostasis, leading to cortisol secretion [8]. Under conditions of prolonged or intense stress, elevated cortisol levels can contribute to the suppression of the immune response, increasing susceptibility to infections and systemic inflammation. Assessing cortisol concentrations during the peripartum period is therefore a sensitive indicator of the animal’s physiological stress status and its ability to adapt to the demands of lactation; it also plays a crucial role in coordinating the metabolic adaptations necessary during this transition by promoting lipolysis, gluconeogenesis, and immune modulation [9,10]. Therefore, the assessment of leptin and cortisol may reflect two complementary aspects of adaptation during the peripartum period: energy balance, activation of the HPA axis and the stress response, which may vary from one breed to another and thus provide significant physiological contrasts [5,6]. The peripartum period is also characterized by a transient activation of inflammatory pathways. In cattle and other domestic species, circulating concentrations of pro-inflammatory cytokines, including interleukin-1β (IL-1β), interleukin-6 (IL-6), and tumor necrosis factor-α (TNF-α), may change around parturition [7,8,11,12]. These mediators play a central role in activating the immune response, regulating acute-phase protein synthesis, and modulating energy metabolism [13,14]. In this complex scenario, acute-phase proteins play a pivotal role. Among them, haptoglobin is one of the most sensitive and informative biomarkers of the inflammatory response in cattle. Its hepatic production is strongly induced by key pro-inflammatory cytokines [15], making it a robust indicator of immune activation and physiological or tissue stress. Cytokines reflect the immediate onset of the inflammatory response, whereas haptoglobin captures its systemic consequences by translating inflammatory signals into a measurable hepatic response that remains relatively stable over time. Although endocrine and inflammatory responses during the transition period have been extensively investigated in dairy cattle, comparatively less information is available on how these responses may differ among breeds with contrasting genetic backgrounds, production histories, and adaptive characteristics. In particular, limited information is available regarding the simultaneous temporal assessment of endocrine and inflammatory biomarkers in Modicana and Holstein cows during the peripartum period. A direct comparison between these two breeds may therefore provide useful information on whether their responses to the physiological challenges associated with calving and the onset of lactation differ in timing.

Holstein cattle, intensively selected worldwide for high milk yield, exhibit a physiology largely driven by productive performance, characterized by elevated energy demands and increased susceptibility to metabolic stress during the transition period [16]. Conversely, the Modicana, a native Sicilian breed, shaped by natural selection, displays metabolic resilience, substantial genetic heterogeneity, enhanced homeostatic capacity under variable environmental conditions, and a lower prevalence of metabolic disorders [17,18,19]. They are mainly reared using extensive systems, utilizing pasture during the grazing season and with little or no supplementary concentrated feed. In some cases, particularly when the animals cannot access pasture, semi-intensive rearing systems are also used [20]. Diet has a decisive influence on the quality characteristics of milk, determining its lipid composition and fatty acid profile. Pasture-based diets enrich milk and its derivatives with their characteristic flavors and colors [21]. More broadly, native breeds exhibit adaptive traits such as improved energy regulation, a more controlled inflammatory response, and greater physiological stability during critical life stages [22]. These production characteristics make the breed of particular interest when investigating physiological responses to environmental and nutritional challenges. The contrasting production histories of Holstein and Modicana cattle provide an appropriate context for investigating whether breed-associated differences are reflected in endocrine and inflammatory responses during the peripartum period. However, such differences may also be influenced by production level, nutritional status, body condition, feed intake, and other animal-level factors. Therefore, assessment of circulating biomarkers can provide valuable information on physiological responses, although it should not be considered a direct measure of resilience or adaptive capacity. Accordingly, the aim of this study was to characterize and compare selected endocrine and inflammatory responses in Holstein and Modicana cows during the peripartum period by assessing serum concentrations of leptin, cortisol, haptoglobin, IL-1β, IL-6, and TNF-α. By examining breed- and time-specific patterns in these biomarkers, the study sought to provide preliminary information on differences in physiological responses between a high-producing cosmopolitan breed and a native Sicilian breed during the transition period. These findings may contribute to the development of further research aimed at understanding the biological basis of adaptation in different dairy cattle populations and its potential relevance to sustainable and animal-welfare-oriented production systems.

## 2. Materials and Methods

### 2.1. Study Design and Animal Management

The trial was carried out in accordance with the ARRIVE guidelines and received institutional approval from the Ethical Animal Care and Use Committee of the Department of Veterinary Sciences, University of Messina (Protocol No. 10/2025), on 22 May 2025. The farm owner was previously informed, and consent for animal use was obtained in compliance with the purposes and methods of the research. A total of 20 multiparous cows, including 10 Modicana cows (2–3 years old; mean body weight: 585 ± 105 kg) and 10 Holstein cows (2–3 years old; mean body weight: 765 ± 115 kg), were enrolled in the study during the spring season (May–June 2025) from a cattle farm located in Ragusa, Sicily, Italy (36°47′08.6″ N 14°32′10.2″ E). The cows were enrolled based on predefined eligibility criteria. Only multiparous Modicana and Holstein cows in good health, with expected calving dates falling within the study window, were included. The cows were monitored across the transition period (from −15 ± 3 to +15 ± 3 days relative to parturition). Before enrollment, all cows underwent a clinical examination; hematological and hematochemical analyses were also performed, and all animals were deemed healthy; no clinical abnormalities were detected at any point during the experimental period. Both breeds were maintained on the same farm and received the same diet, as reported in Table 1. In addition, the cows had access to pasture for approximately 6 h/day.

### 2.2. Environmental Conditions

**Figure 1:**
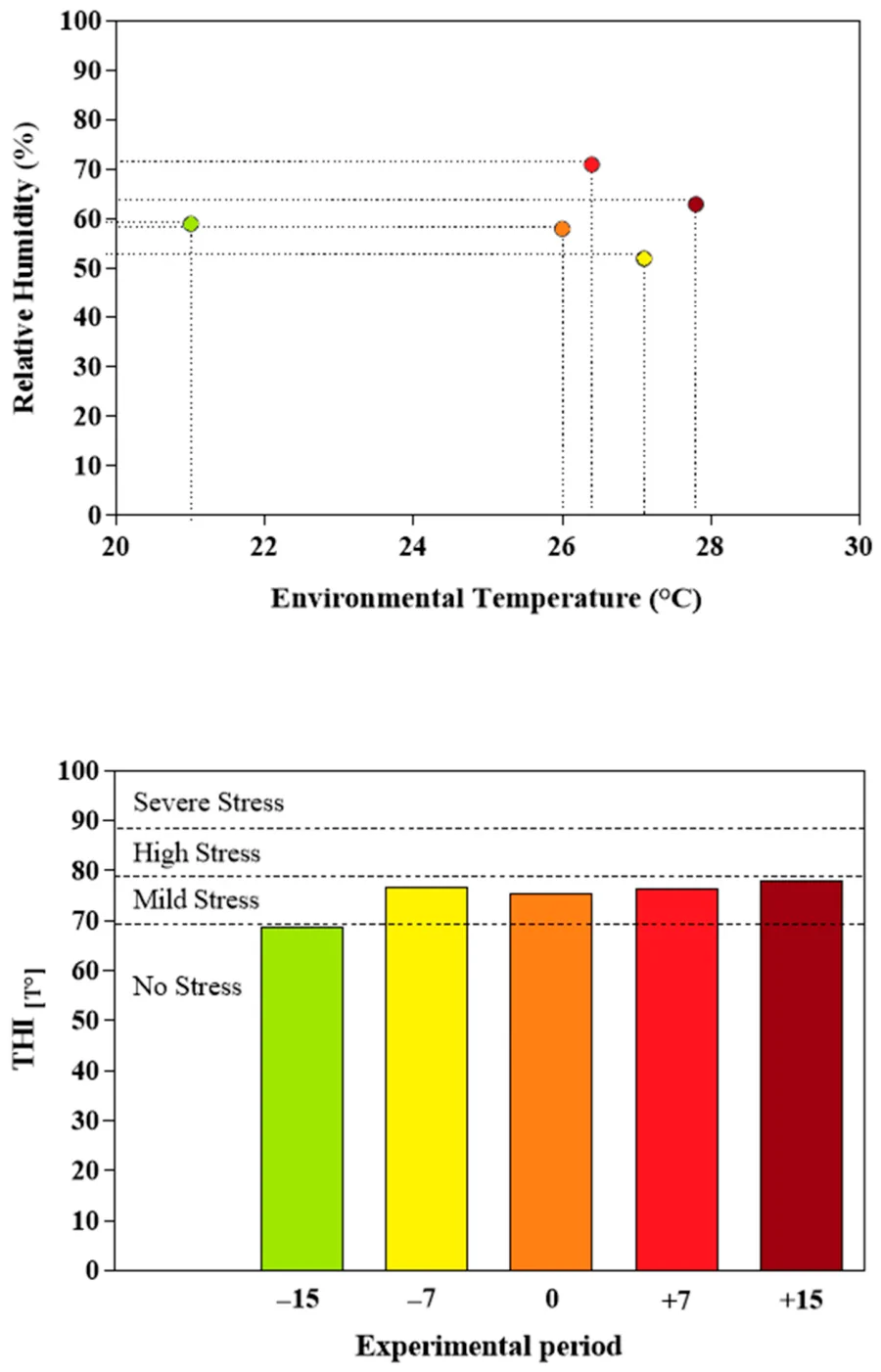
Mean ambient temperature (°C), relative humidity (RH, %), and Temperature–Humidity Index (THI) recorded during the experimental period at −15, −7, 0, +7, and +15 days relative to parturition. Sampling times are distinguished by colors: green (−15 d), yellow (−7 d), orange (0 d), red (+7 d), and burgundy (+15 d).

Environmental conditions recorded during the experimental period are presented in Figure 1. Ambient temperature (°C) and relative humidity (RH%) were continuously monitored throughout the study using a high-accuracy, high-resolution multiparameter probe (Testo 400; Testo SE & Co. KGaA, Lenzkirch, Germany) installed inside the stall. Mean ambient temperature and relative humidity values recorded by the data logger were used to calculate the Temperature–Humidity Index (THI). THI, commonly used as an indicator of thermal comfort and heat stress in cows, was calculated according to the U.S. Weather Bureau Temperature–Humidity Index formula for bovine species [23].

THI [°C] = T°ambient + (0.36 ∗ dew-point temperature) + 41.5

### 2.3. Blood Sample Collection and Laboratory Analysis

Blood samples were collected by caudal vein into a 9 mL tube with a clot activator (Vacutainer^®^, Becton Dickinson, Franklin Lakes, NJ, USA). The samples were collected 15 ± 3, 7 ± 3 days before expected calving (−15 d, −7 d), within 4 h after calving (0), and 7 ± 3, 15 ± 3 days after calving (+7 d, +15 d). After blood collection, all samples were immediately stored in a cooler. The tubes were centrifuged at 1900× *g* for 16 min at 4 °C. Serum was aliquoted and stored at −20 °C until analysis. Serum concentrations of leptin, IL-1β, IL-6, TNFα, cortisol and haptoglobin were determined using ELISA kits specific for bovine species. Leptin was measured using a Bovine Leptin ELISA kit (MyBioSource, Inc., San Diego, CA, USA, Cat. No. MBS703026; sensitivity 3.12 ng/mL; the intra- and the inter-assay coefficients of variation were <15); IL-1β was measured using a Bovine Interleukin-1 beta (IL-1β) ELISA kit (MyBioSource, Inc., San Diego, CA, USA, Cat. No. MBS1602208; sensitivity 0.25 ng/L; the intra- and the inter-assay coefficients of variation were <8 and <10%, respectively); IL-6 was measured using a Bovine Interleukin-6 (IL-6) ELISA kit (MyBioSource, Inc., San Diego, CA, USA, Cat. No. MBS1602208; sensitivity 2.0 pg/mL; the intra- and the inter-assay coefficients of variation were <15%); TNF-α was measured using a Bovine Tumor Necrosis Factor-α (TNF-α) (MyBioSource, Inc., San Diego, CA, USA, Cat. No. MBS2707990; sensitivity 2.8 pg/mL; the intra-and the inter-assay coefficients of variation were <10 and <12% respectively). Cortisol was measured using a Cortisol ELISA kit (Elabscience^®^, Houston, TX, USA, Cat No. E-EL-0158; sensitivity 0.96 ng/mL; the intra- and the inter-assay coefficients of variation were <10%). Haptoglobin was measured using the PHASE™ RANGE Haptoglobin kit (Tridelta Ltd., Maynooth, Ireland; sensitivity 0.005 mg/mL; the intra- and inter-assay coefficients of variation were <7% and <6%). All assays were performed using a microtiter plate reader (BK−EL10B, Biobase Biodustry Co., Ltd., Jinan, China). All calibrators and samples were run in duplicate, and the samples exhibited parallel displacement to the standard curve for all ELISA analyses.

### 2.4. Statistical Analysis

The data were analyzed with the software Prism v. 9.00 (GraphPad Software Ltd., San Diego, CA, USA). The normal distribution of the data was verified by the application of the Shapiro–Wilk test. The data were normally distributed (*p* > 0.05). A two-way repeated-measures analysis of variance (ANOVA) was performed, with breed included as the between-subject factor and sampling time as the repeated within-subject factor. The breed × time interaction was also tested to assess potential differences in temporal responses between breeds. The assumptions of the repeated-measures analysis were evaluated using residual diagnostics and Mauchly’s test for sphericity. When the assumption of sphericity was violated, the Greenhouse–Geisser correction was applied. Tukey’s multiple comparison test was applied for post hoc mean comparison for investigating differences between breeds. The Pearson correlation analysis was applied to assess the possible correlation between the concentration of cortisol and/or haptoglobin and interleukins obtained in both cattle breeds. The Pearson correlation analyses were performed separately for Holstein and Modicana cows at each sampling time (−15, −7, 0, +7, +15 days). For each correlation, all animals were included (n = 10 per breed), with no missing values or exclusions. A total of 80 correlations were tested: 30 correlations between cortisol and the three interleukins (IL-6, IL-1β, TNF-α) and 50 correlations between haptoglobin and all parameters tested (leptin, cortisol, IL-6, IL-1β, TNF-α) across both breeds and all sampling times. *p* values < 0.05 were considered statistically significant.

## 3. Results

The results are expressed as mean values ± standard error of the mean (±SEM).

**Figure 2:**
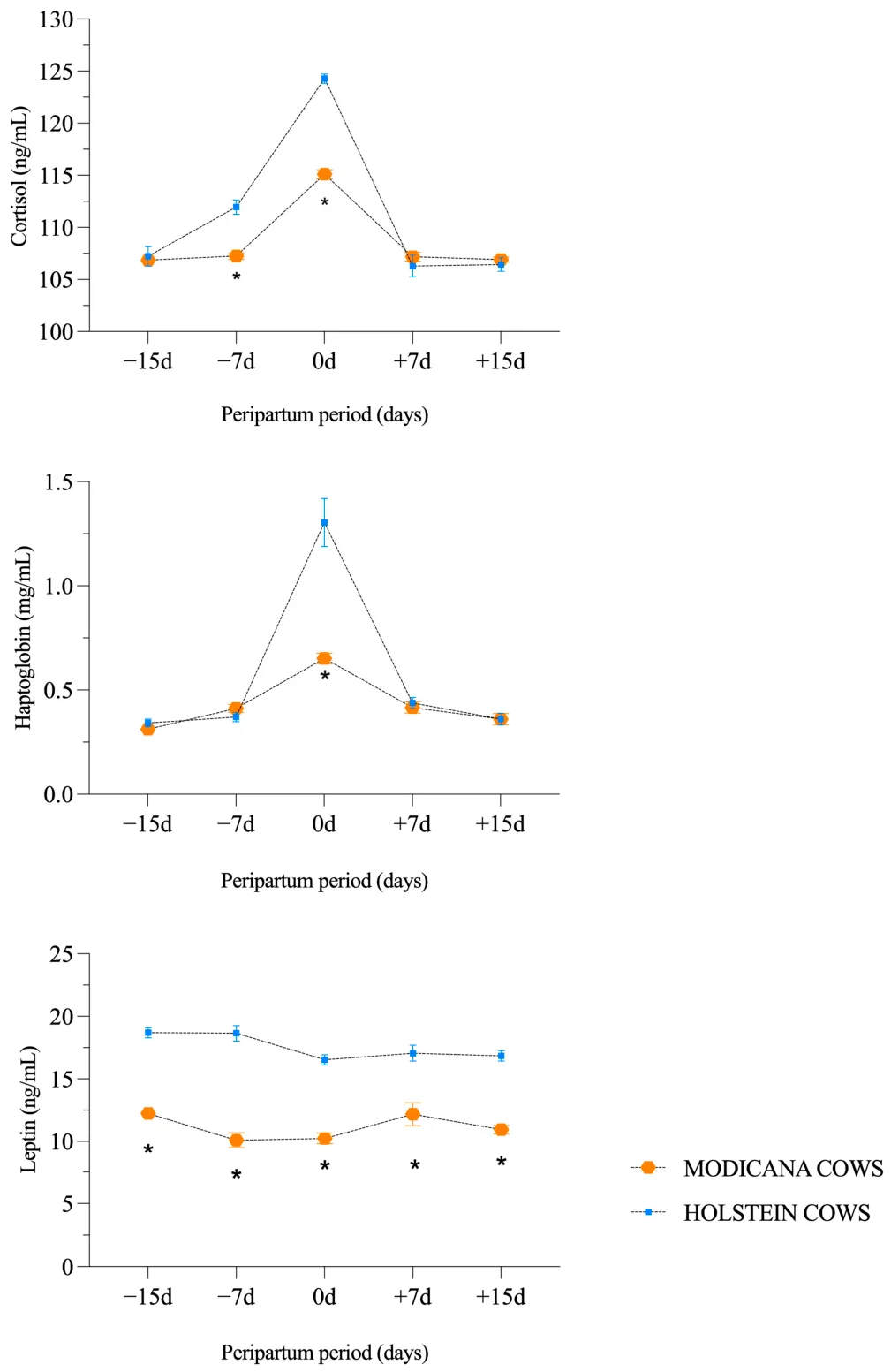
Mean values ± standard error of the mean (±SEM) of serum cortisol, haptoglobin and leptin measured between breeds during experimental period (15 days before calving, −15 d; 7 days before calving day, −7 d; calving day, 0; 7 days after calving, +7 d; 15 days after calving, +15 d), together with the corresponding statistical significances. Significant differences between breeds (*p* < 0.0001) are indicated by * relative to Holstein cows.

**Figure 3:**
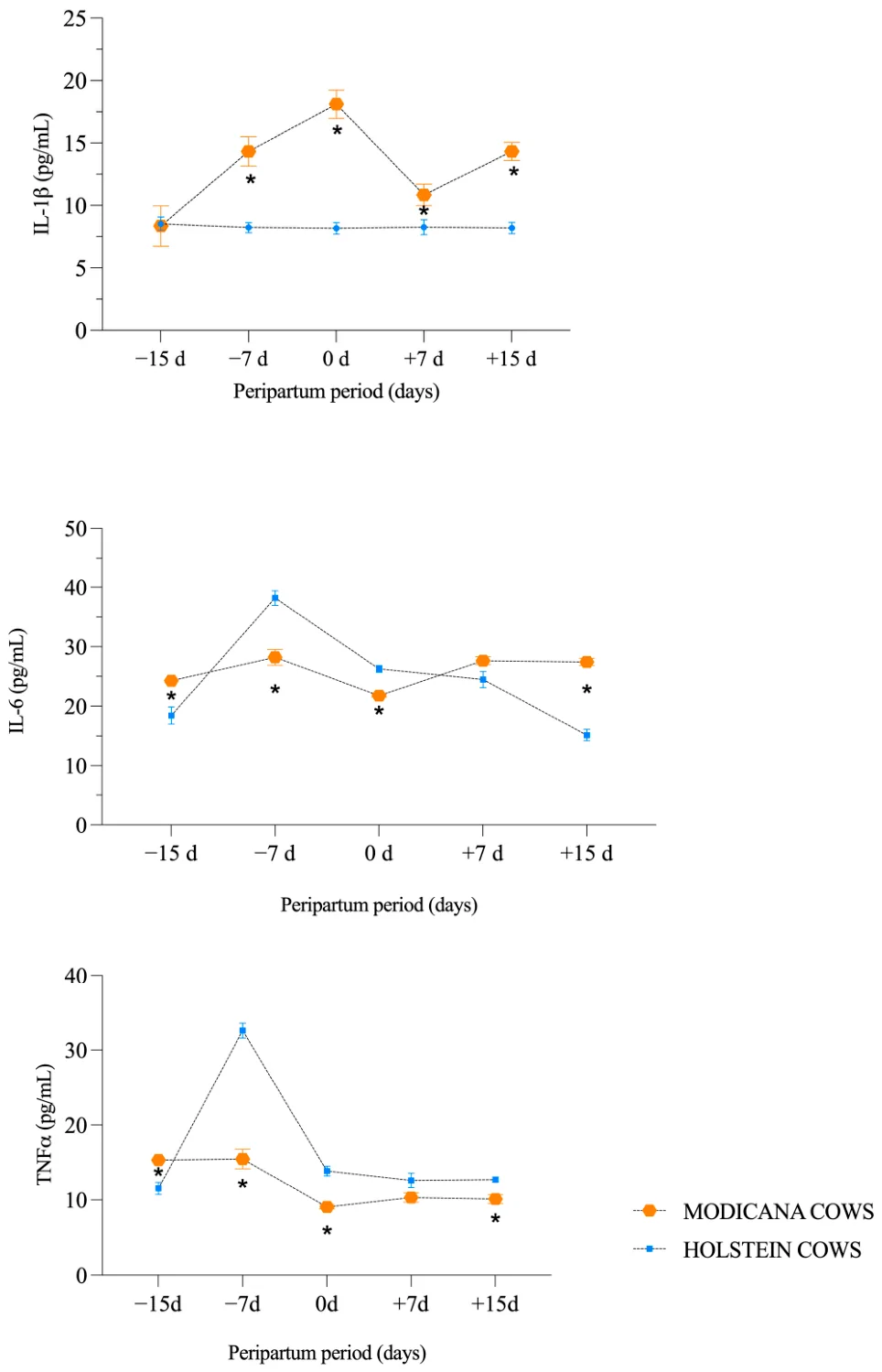
Mean values ± standard error of the mean (±SEM) of serum interleukin (IL)-1β, interleukin (IL)-6, tumor necrosis factor-α (TNFα) measured between breeds during the experimental period (15 days before calving, −15 d; 7 days before calving day, −7 d; calving day, 0; 7 days after calving, +7 d; 15 days after calving, +15 d), together with the corresponding statistical significances. Significant differences between breeds (*p* < 0.0001) are indicated by * relative to Holstein cows.

During the experimental period, the Temperature–Humidity Index (THI) progressively increased from −15 d to +15 d, reaching the highest value at +15 d (Figure 1). Both the Modicana and Holstein cows showed the highest serum cortisol concentration at calving (0 d; *p* < 0.0001; Table 2). In addition, in the Holstein cows, cortisol levels were also higher at −7 d than at +7 d (*p* < 0.0001; Table 2). Haptoglobin concentration was highest at calving in both the Modicana and Holstein cows compared with other time points (*p* < 0.0001; Table 2). Only in the Holstein cows did the results show that haptoglobin levels were highest at +7 d compared with −15 d (*p* < 0.0001; Table 2). The investigated Modicana cows showed higher levels of leptin at −15 d than −7 d (*p* < 0.0001; Table 2). In the Holstein cows the concentration of leptin was higher at −7 d than 0 d (*p* < 0.0001; Table 2). The results for IL-1β concentration in the Modicana breed showed the highest levels at 0 d compared with −15 d and +7 d. In addition, the levels of IL-1β were found to be high at −7 d compared to −15 d (*p* < 0.0001; Table 2). In Modicana cows, the highest IL-6 levels were detected at +7 d compared with 0 d and −15 d. The lowest concentration of IL-6 was detected at 0 d compared with −15 d, −7 d and +15 d. IL-6 levels were higher at +15 days than at −15 days (*p* < 0.0001; Table 2). In the Holstein breed, the highest levels of IL-6 were detected at −7 d compared with other time points. The lowest levels of IL-6 were detected at +15 d compared with 0 d and +7 d. The IL-6 levels detected were lower at −15 days than at 0 days (*p* < 0.0001; Table 2). In the Modicana cows, the highest levels of TNFα were detected at −7 d (*p* < 0.0001; Table 2). In the investigated Holstein cows, the levels of TNFα were higher at −7 d compared to the other time points tested (*p* < 0.0001; Table 2). Post hoc between-breed comparisons showed that the Holstein cows had higher cortisol levels at −7 days (*p* < 0.0001; Figure 2) and at 0 d (*p* < 0.0001; Figure 2) compared with Modicana cows. For haptoglobin, a significant breed effect was observed only at 0 d (*p* < 0.0001; Figure 2), with higher concentrations recorded in Holstein cows. Regarding leptin, Holstein cows exhibited at all sampling points consistently higher serum concentrations than Modicana cows (*p* < 0.001; Figure 2). For IL-1β, Modicana cows displayed higher IL-1β concentrations compared with Holstein cows at all sampling points (*p* < 0.001; Figure 3), with the exception of −15 d, where no significant difference was found (*p* > 0.05; Figure 3). Significant between-breed differences were also found for IL-6 and TNFα; the Holstein cows showed higher concentrations of IL−6 at −7 d, 0 d than the Modicana breed and the lowest levels at −15 d and +15 d (*p* < 0.001; Figure 3). TNFα levels were higher in the Modicana cows than in the Holstein cows at −15 d (*p* < 0.001; Figure 3). However, at −7 d, 0 d, and +15 d, TNFα levels were higher in the Holstein cows than in the Modicana cows (*p* < 0.001; Figure 3). Regarding the correlation analysis, serum cortisol concentration showed a significant negative correlation with IL-6 in Holstein cows at −15 d (r = −0.73; *p* = 0.01). This correlation was based on 10 observations, with all animals included and no missing values or exclusions. No other significant correlations were detected among the variables analyzed. The complete results of the repeated-measures ANOVA (effect of breed, time, and breed for time interaction) are presented in Table 3.

## 4. Discussion

The peripartum period represents one of the most physiologically demanding phases for dairy cattle, requiring extensive endocrine, metabolic, and immune adaptations to support fetal maturation, parturition, placental expulsion, and the onset of lactation [24]. Although these processes have been extensively investigated in high-yielding dairy breeds, information regarding breed-specific adaptive responses remains limited, particularly in native breeds such as Modicana. The present study contributes to comparing endocrine and inflammatory responses in Modicana and Holstein cows during the transition period, providing insight into potential differences in the temporal patterns of selected physiological biomarkers. The cortisol patterns in both breeds, marked by peak concentrations at calving, reflect activation of the hypothalamic–pituitary–adrenal axis during the peripartum period. This endocrine response supports the physiological demands of parturition by coordinating energy metabolism, mobilizing nutrient reserves, and modulating immune function [25,26]. Beyond its metabolic functions, cortisol also contributes to controlling physiological inflammation, thereby regulating the inflammatory processes [2]. The higher cortisol concentrations observed in Holstein cows at −7 d and at calving indicate a greater activation of the HPA axis response at these specific time points. However, cortisol concentrations can be influenced by several physiological and environmental factors, and the present results should therefore be interpreted as differences in endocrine responses rather than direct evidence of greater stress or reduced welfare in Holstein cows. The simultaneous increase in haptoglobin concentrations further supports the existence of a coordinated endocrine–immune response during the transition period. Haptoglobin is one of the major acute-phase proteins in cattle and is widely recognized as an indicator of inflammatory activation [15,27]. The transient rise observed around calving in both breeds most likely reflects the physiological inflammatory response that accompanies the peripartum period; these findings confirm that activation of the acute-phase response is a normal component of the periparturient adaptation process and occurs in conjunction with endocrine changes aimed at maintaining homeostasis. Leptin concentrations were highest during late gestation and remained generally greater in Holstein cows throughout the experimental period. As an adipokine involved in the regulation of energy balance, leptin also exerts immunomodulatory actions and contributes to cytokine regulation, thereby functioning as a biological link between metabolic and inflammatory pathways [28,29]. The higher leptin concentrations observed in Holstein cows may therefore indicate differences in metabolic or endocrine status between the two groups. Nevertheless, body condition score, adiposity, individual feed intake, milk production, and detailed metabolic indicators were not assessed in the present study [29].

The cytokine profiles further support the presence of physiological inflammatory activation during the transition period. This interpretation is consistent with the roles of IL-1β, IL-6, and TNF-α, which contribute to the regulation of innate immune responses, tissue remodeling, and acute-phase protein synthesis [20,21,22,23,24,25,26,27,28,29,30,31]. However, the three cytokines showed distinct temporal and breed-specific patterns in the present study, but all the cytokine profiles further support the occurrence of physiological inflammatory activation during the transition period. IL-1β, IL-6, and TNF-α are involved in the regulation of innate immune responses, tissue remodeling, and acute-phase protein synthesis [30,31]. IL-6 concentrations were higher in Holstein cows at −7 and 0 d, whereas TNF-α concentrations were higher in Modicana cows at −15 d but higher in Holstein cows at −7, 0, and +15 d. These findings indicate that breed differences were not uniform across inflammatory biomarkers and should therefore be interpreted on a cytokine-specific basis. IL-1β showed a pattern that differed from that observed for the other inflammatory biomarkers; in fact Modicana cows showed higher IL-1β concentrations than Holstein cows at −7, 0, +7, and +15 d, while no significant between-breed difference was observed at −15 d. The higher concentrations observed in Modicana cows at most sampling times may indicate differences in the magnitude or timing of IL-1β-related inflammatory signaling. The IL-1β profile therefore further highlights the complexity of inflammatory regulation during the transition period, rather than indicating a uniform response across all cytokines; indeed, it appears that Modicana cows exhibit a more rapid early innate immune activation, which is reflected in their higher concentrations of IL-1β. Unlike IL-6 and TNF-α, IL-1β is primarily driven by local and immediate inflammatory stimuli rather than by metabolic load [26]. Consequently, Modicana cows may trigger the early inflammatory cascade more rapidly, whilst Holstein cows could exhibit a later and more systemic cytokine response [24].

Holstein cows generally displayed higher cortisol, haptoglobin, leptin, and IL-6 concentrations at specific sampling times, and the responses observed in Holstein cows may reflect the physiological demands associated with intensive genetic selection for milk production and the significant metabolic challenges that high-producing cows face during the transition period. Holstein cows have undergone decades of intensive selection aimed at high milk production [32,33], a process that has progressively increased stress during the transition period.

However, milk production was not individually quantified in the present study, and therefore its potential contribution to the observed between-breed differences could not be formally assessed. Similarly, individual feed intake, body condition score, energy balance, and detailed metabolic status were not measured. Consequently, the observed differences cannot be attributed directly to differences in milk production or production-related metabolic demands. The different genetic and productive histories of the two breeds may nevertheless provide a possible context for interpreting the observed patterns. Holstein cattle have been intensively selected for milk production, whereas Modicana cattle represent a native Sicilian breed whose production system and breeding history have been more closely associated with adaptation to local environmental and management conditions [17,19]. These differences may contribute to variation in physiological responses during challenging periods. However, the present study cannot determine whether the observed biomarker patterns are directly attributable to genetic background, production level, management history, or interactions among these factors. The concept of greater allostatic load should therefore be interpreted cautiously. Although the higher concentrations of some stress- and inflammation-related biomarkers in Holstein cows could be compatible with greater physiological demands, allostatic load was not directly assessed in this study. Considering that 80 Pearson correlations were performed, the correlation analysis—including the cortisol–IL-6 association—should be interpreted as exploratory, but it can be considered interesting that a negative correlation was observed between cortisol and IL-6 concentrations at 15 days before calving in Holstein cows. This relationship may reflect the immunomodulatory effects of glucocorticoids [34], but this correlation alone does not provide sufficient evidence for tighter endocrine control of inflammation or for different endocrine–immune regulatory mechanisms between breeds, and further studies involving larger sample sizes and repeated measurements are required to clarify its biological significance. Differences between breeds were also evident during the postpartum period, although their direction varied according to the specific cytokine and sampling time. Therefore, the results do not support a general statement that Modicana cows showed higher cytokine concentrations two weeks after parturition. Instead, the observed patterns suggest that the timing of individual inflammatory responses differed between the two groups; whether these differences are associated with differences in the rate of physiological recovery cannot be established from the present data, as metabolic, productive, health, and reproductive recovery were not directly assessed.

Environmental conditions recorded during the experimental period showed an overall increase in THI, with the highest value recorded at +15 d (Figure 1).

Both breeds were maintained under the same management, nutritional, and environmental conditions. Therefore, environmental exposure was broadly comparable between the two groups. Nevertheless, individual differences in pasture intake and other animal-level factors could not be excluded and may have contributed to variation in biomarker concentrations. The present findings therefore support the finding that Holstein and Modicana cows exhibit different biomarker response patterns during the peripartum period, rather than clearly demonstrating distinct adaptive strategies. Holstein cows showed greater concentrations of several biomarkers at specific time points, whereas Modicana cows showed a more moderate response for some of the parameters investigated. However, these findings should not be interpreted as direct evidence that Modicana cows possess greater physiological resilience. Physiological resilience is a complex characteristic that requires the integration of metabolic, productive, health, reproductive, and behavioral outcomes, which were not directly assessed in the present study. The observed differences may nevertheless provide a basis for further investigation of how native and highly selected dairy populations respond to the physiological challenges of the transition period. In particular, the potential adaptive characteristics of Modicana cattle should be evaluated using larger populations and multiple herds, together with integrated endocrine, inflammatory, metabolic, productive, and health indicators. Such studies may help clarify whether the biomarker patterns observed here are associated with meaningful differences in adaptive capacity and resilience.

A limitation of the present study is the relatively limited number of animals and the fact that observations were conducted under a single management system. A formal sample size calculation was not performed.

The number of animals included (n = 10 per breed) reflected the availability of eligible cows under uniform management conditions. This limited sample size may reduce the ability to detect small effect sizes and should therefore be considered a limitation of the study. Consequently, caution should be exercised when extrapolating these findings to broader populations. Furthermore, individual pasture intake was not directly measured, and milk production, body condition score, and additional metabolic indicators were not systematically assessed. The absence of additional metabolic indicators, such as non-esterified fatty acids and β-hydroxybutyrate, limits the possibility of fully characterizing the metabolic adaptations associated with the observed stress and inflammatory responses. Finally, because Holstein and Modicana cows differ not only in genetic background but also in production level and metabolic demands, it is difficult to completely disentangle breed effects from production-related or other animal-level factors. This constraint should be considered when interpreting the between-breed differences reported in the present study.

Overall, the present results provide preliminary evidence of breed- and time-dependent differences in selected endocrine and inflammatory biomarkers during the peripartum period. While these findings are compatible with differences in the physiological responses of Modicana and Holstein cows, further research is needed to determine whether such differences translate into distinct adaptive capacities or resilience under different production and environmental conditions.

## 5. Conclusions

This study identified breed- and time-dependent differences in selected endocrine and inflammatory biomarkers during the peripartum period in Modicana and Holstein cows reared under the same general environmental and management conditions. Holstein cows showed higher concentrations of cortisol, haptoglobin, leptin, and IL-6 and a higher level of IL-1β in Modicana cows at specific sampling times. Consequently, Modicana cows may trigger the early inflammatory cascade more rapidly, whilst Holstein cows could exhibit a later and more systemic cytokine response. These findings indicate differences in temporal dynamics of endocrine and inflammatory responses between the two groups. The observed biomarker patterns may be compatible with differences in the physiological responses of the two breeds during the transition period. However, the present study does not allow us to directly assess physiological resilience or to determine whether the observed differences are attributable to breed, production level, metabolic status, or other animal-level factors. Therefore, the findings should be considered preliminary and interpreted with caution, particularly given the limited sample size and the single-farm design. Further studies involving larger populations and multiple farms, together with the assessment of milk production, body condition, feed intake, metabolic, health, and reproductive indicators, are warranted to clarify the biological significance of these breed-specific responses and to determine their potential relevance to adaptation and resilience in dairy cattle.

## Figures and Tables

**Table 1:** Feed ingredients and chemical composition of the diets provided to Modicana and Holstein cows before and after calving.

Feed Ingredients (kg/Day per Head)	Before Calving	After Calving
Fresh pasture ^1^	5.00	4.00
Grass hay	2.50	2.00
Corn silage	6.00	8.00
Corn grain	0.80	2.00
Barley grain	0.80	1.20
Soybean meal	0.30	0.70
Wheat bran	0.70	1.00
Beet pulp (dry)	–	0.80
Mineral–vitamin premix	0.15	0.20
Total	16.25	19.90
Chemical Composition		
Dry matter (%)	42.5	44.0
Crude protein (%)	14.2	16.2
Ether extract (%)	3.3	4.2
Crude fiber (%)	19.0	17.0
Ash (%)	7.0	7.2
NDF (%)	47.0	41.0
ADF (%)	29.0	25.0
Starch (%)	14.0	21.0
Net energy for lactation (Mcal/kg DM)	1.40	1.56
Calcium (g/day) ^2^	72	98
Phosphorus (g/day) ^2^	31	42

^1^ Pasture intake represents an estimated value and was not individually measured for each cow; ^2^ Calcium and phosphorus values represent estimated daily intakes calculated from the amount of each feed ingredient supplied and its respective mineral content.

**Table 2:** Mean values ± standard error of the mean (±SEM) of the parameters measured in the Modicana and Holstein cows during the experimental period (15 days before calving, −15 d; 7 days before calving day, −7 d; calving day, 0; 7 days after calving, +7 d; 15 days after calving, +15 d), together with the corresponding statistical significances related to the peripartum period.

Parameters		Peripartum Period				
	Cattle Breed	−15 d	−7 d	0 d	+7 d	+15 d
**Cortisol (ng/mL)**	Modicana	106.86 ± 0.49 ^c^	107.26 ± 0.98 ^c^	115.11 ± 0.44	107.19 ± 0.42 ^c^	106.92 ± 0.23 ^c^
	Holstein	107.23 ± 0.93 ^b,c^	111.96 ± 0.69 ^c^	124.27 ± 0.43	106.30 ± 1.04 ^b,c^	106.44 ± 0.64 ^b,c^
**Haptoglobin (mg/mL)**	Modicana	0.31 ± 0.03 ^c^	0.41 ± 0.02 ^c^	0.65 ± 0.03	0.41 ± 0.03 ^c^	0.36 ± 0.03 ^c^
	Holstein	0.34 ± 0.02 ^c^	0.37 ± 0.02 ^c^	1.30 ± 0.12	0.44 ± 0.03 ^a,c^	0.36 ± 0.03 ^c^
**Leptin (ng/mL)**	Modicana	12.25 ± 0.55 ^b^	10.10 ± 0.60	10.24 ± 0.42	12.17 ± 0.92	10.94 ± 0.35
	Holstein	18.69 ± 0.41	18.65 ± 0.62 ^c^	16.53 ± 0.41	17.06 ± 0.63	16.84 ± 0.42
**IL** **-** **1β (pg/mL)**	Modicana	8.36 ± 1.61 ^c^	14.33 ± 1.18	18.11 ± 1.13	10.84 ± 0.85 ^c^	14.32 ± 0.73
	Holstein	8.54 ± 0.53	8.23 ± 0.40	8.17 ± 1.49	8.26 ± 0.60	8.20 ± 0.45
**IL** **-** **6 (pg/mL)**	Modicana	24.28 ± 0.36 ^c^	28.21 ± 1.31 ^c^	21.79 ± 0.28	27.65 ± 0.64 ^a,c^	27.45 ± 0.60 ^a,c^
	Holstein	18.45 ± 1.45 ^b,c^	38.17 ± 1.23 ^d^	26.25 ± 0.58 ^b^	24.48 ± 1.33	15.16 ± 0.94 ^b,c,d^
**TNF** **-** **α (pg/mL)**	Modicana	15.36 ± 0.36 ^c^	15.48 ± 1.31 ^c^	9.06 ± 0.28	10.32 ± 0.64 ^a,b^	10.12 ± 1.13 ^a,b^
	Holstein	11.56 ± 0.80 ^b^	32.57 ± 6.15 ^c^	13.88 ± 0.68	12.61 ± 0.96 ^b^	12.73 ± 0.34 ^b^

Significant effect of peripartum period (*p* < 0.05): ^a^ vs. −15 d; ^b^ vs. −7 d; ^c^ vs. 0; ^d^ vs. +7.

**Table 3:** F-values and *p*-values of all biomarkers tested for the effect of breed and time and the breed × time interaction.

Biomarkers	Breed Effect (F and *p* Value)	Time Effects (F and *p* Value)	Breed × Time Interaction (F and *p* Value)
Cortisol (ng/mL)	F = 51.05; *p* < 0.0001	F = 158.9; *p* < 0.0001	F= 23.71; *p* < 0.0001
Haptoglobin(mg/mL)	F = 18.94; *p* < 0.0004	F = 82.77; *p* = 0.0001	F = 23.95; *p* < 0.0001
Leptin (ng/mL)	F = 363.3; *p* < 0.0001	F = 3.89; *p* = 0.0176	F = 2.82; *p* = 0.031
IL-1β (pg/mL)	F = 52.02; *p* < 0.0001	F = 9.52; *p* < 0.0001	F = 10.91; *p* < 0.0001
IL-6 (pg/mL)	F = 5.76; *p* = 0.02	F = 49.92; *p* < 0.0001	F = 39.65; *p* < 0.0001
TNF-α (pg/mL)	F = 108.0; *p* < 0.0001	F = 98.52; *p* < 0.0001	F = 48.53; *p* < 0.0001

## Data Availability

The original contributions presented in this study are included in the article.
